# An Exploration of the Content and Language Used in Publicly Available National Health Service Patient Information Leaflets for People Considering Shoulder Replacement Surgery: A Qualitative Study

**DOI:** 10.1002/msc.70079

**Published:** 2025-03-10

**Authors:** Maria Moffatt, Nina Chalmers, Chris Littlewood

**Affiliations:** ^1^ University of Liverpool Liverpool England; ^2^ St. John's Hospital NHS Lothian Livingston Scotland; ^3^ University of Salford Salford England

**Keywords:** health communication, NHS patient education, patient information leaflets, shared decision making, shoulder replacement surgery

## Abstract

**Objectives:**

The decision to undergo total shoulder replacement surgery is a major one and should be a joint one between the patient and surgeon. It is important that patients are provided with accessible, meaningful and appropriate information to enable an informed decision. The aim of this study was to explore the content and language used within publicly available information leaflets produced by UK National Health Service (NHS) Trusts for people considering shoulder replacement surgery and to consider how this may influence surgical decision making.

**Design:**

An online search of publicly available NHS shoulder replacement patient information leaflets (PIL) was undertaken. The text within the PIL was analysed using reflexive thematic analysis.

**Results:**

Thirty‐eight PIL were identified. The volume of information and content varied greatly. All PIL discussed the clinical problem, mainly within a biomedical framework and from a clinician's perspective in which normal shoulder anatomy was contrasted with shoulder pathology. Only a minority of the PIL discussed non‐surgical treatments and of those that did, such approaches were predominantly portrayed as a temporary management option only, whilst surgery was frequently portrayed as the optimum treatment.

**Conclusion:**

There is variation in the content of NHS shoulder replacement PIL. The content and language used may not adequately support people in making an informed decision about whether surgery is the right treatment option for them. We need to better understand the information needs of people considering shoulder replacement surgery, and provide information that is accessible, culturally sensitive, and capable of facilitating shared decision making.

## 1 Introduction

Shoulder replacement surgery is one treatment option for people with shoulder osteoarthritis when pain, stiffness and functional impairments are significantly affecting quality of life and have not improved with non‐surgical management (National Institute for Health and Care Excellence (NICE), 2022).

The decision to undergo shoulder replacement surgery is a major one (Davies et al. 2024) and should be shared between a patient and surgeon considering individual patients' priorities, values, and circumstances (NICE 2021). To enable shared decision making, patients should be provided with adequate information regarding risks, benefits, natural history, and alternative treatments (Elwyn et al. 2017). Discussions that take place during healthcare consultations are recognised as the main source of health information provision for patients (Kennedy et al. 2017); however, these can be challenging due to limited clinic time (Kennedy et al. 2017; Mira et al. 2014) and the fact that a high proportion of information conveyed in this context is not retained (Menichetti et al. 2021). To complement clinical consultations, written material can help inform patients about their condition and the available treatment options (Giguère et al. 2020; Menichetti et al. 2021); therefore, NHS Trusts frequently develop and publish patient information leaflets (PILs). To achieve their aim, it is important that PILs are accessible, meaningful, and understandable (Findeis and Patyk 2020). Hence, the aim of this study was to explore the language and content used within publicly available PILs produced by the National Health Service (NHS) in the United Kingdom (UK) regarding shoulder replacement surgery and consider how these might influence patient decision making.

## 2 Methods

As this was a study of publicly available information and no participants were involved, ethical approval was not required. The completed COREQ checklist is available as a supplementary file in Supporting Information S1.

### 2.1 Data Collection

PIL were identified through an online search of Google up to 09 December 2024 using the following search terms:

- Shoulder replacement NHS
- Shoulder arthroplasty NHS
- Shoulder replacement surgery NHS
- Shoulder arthroplasty surgery NHS
- Shoulder replacement information leaflet NHS
- Shoulder arthroplasty information leaflet NHS

We searched the first five pages of the Google search results for each search term to ensure comprehensiveness, recognising that only 7% of people search beyond the first page (Ray 2019). Searches were carried out by one author (NC) and updated and verified by a second author (CL).

### 2.2 Inclusion Criteria

PIL were included if they were published by UK NHS Trusts and publicly available, and provided information about shoulder replacement surgery (anatomic total shoulder replacement, reverse total shoulder replacement, shoulder hemi‐arthroplasty, and shoulder resurfacing) including the nature of the procedure, clinical indications for surgery, risks, alternative treatment options, and prognosis.

### 2.3 Exclusion Criteria

PIL were excluded if they did not contain any detail about shoulder replacement surgery, for example, if they include only postoperative protocols.

### 2.4 Data Analysis and Reflexivity

All textual information that aligned with the study aims was extracted and exported into Microsoft Excel (2020). Data were analysed using the reflexive thematic analysis approach as described by Braun and Clarke (Braun and Clarke 2023; Byrne 2022). This method of qualitative data analysis is used to identify patterns within data and provides a method to describe and interpret the meaning of these patterns (Clarke and Braun 2017). It was selected to enable an in‐depth exploration of the content of the PIL, whereby codes and themes were developed inductively from the extracted data as opposed to using a pre‐existing thematic framework. Reflexive thematic analysis involves six stages including familiarisation, generating initial codes, searching for themes, reviewing themes, defining and naming themes, and then producing the report (Braun and Clarke 2023; Clarke and Braun 2017).

Data were initially coded by one author (NC); the list of codes was then reflexively discussed among the research team until a comprehensive coding framework was finalised; this was then applied to the entire dataset. The coded data were then reviewed by the research team, and all authors were involved in developing, refining, and naming the themes.

MM is a female post‐doctoral academic physiotherapist with experience of qualitative research. NC is a female clinical physiotherapist qualified to Master's level with prior experience of qualitative data analysis. CL is a male Professor of rehabilitation with extensive experience of both qualitative and quantitative research. All three members of the research team have an interest in shoulder rehabilitation and shared decision making.

### 2.5 Patient and Public Involvement

This study was inspired by clinical discussions with patients awaiting shoulder replacement surgery. However, patient representatives and members of the public were not involved in the data analysis or reporting of this manuscript.

## 3 Results

A total of 38 PIL from 27 of the UK's 215 NHS trusts were retrieved. These ranged in publication date from 2015 to 2024, with 25/38 published in the last 3 years. Five PIL did not specify a publication date. The content varied greatly as did the volume of information, with PILs ranging in length from 3 to 44 pages.

Two predominant themes, each with two subthemes, were developed from the data in relation to the aims of the study:

1. Framing the clinical problem
  - The ‘normal’ shoulder
  - The osteoarthritic shoulder
2. Surgery as the optimum treatment
  - Alternatives to surgery
  - Surgical decision making

### 3.1 Theme 1: Framing the Clinical Problem

All PIL (38/38) framed the clinical problem, with the majority describing the anatomy of the normal shoulder in comparison to the osteoarthritic shoulder. The level of detail varied greatly between PIL.

### 3.2 Subtheme 1.1 The ‘Normal’ Shoulder

The PILs described the anatomy of the shoulder, predominantly in relation to the glenohumeral joint, most commonly describing it as a ball and socket joint. Some PILs provided further detail including an explanation of the range of movement and function available at the shoulder:

The shoulder joint is a ball and socket joint. Most shoulder movement occurs where the ball at the top of your arm bone (the humerus) fits into the socket (the glenoid), which is part of the shoulder blade (the scapula). (PIL12)

The importance of normal shoulder anatomy was explained in several PILs, with some recognising the role the rotator cuff plays in providing stability to the shoulder:

Some of the movement and stability of the shoulder comes from the rotator cuff muscles. The rotator cuff is the name given to the four main muscles/tendons which play a very important role in keeping your shoulder joint in the right position, enabling you to move your shoulder and arm up and down. (PIL 3)

Consistently, PILs highlighted the importance of the articular cartilage in protecting the bones and allowing smooth shoulder movement:

The surfaces of the bones where they touch (articulate) are covered with cartilage; which protects the bones and allows them to move easily. In a healthy shoulder there is a small amount of fluid that lubricates the cartilage and reduces friction within the shoulder. (PIL 7)

These descriptions might risk inferring that the anatomy of the shoulder must be without defects to function without pain, which does not align with contemporary understanding (NICE, 2022).

### 3.3 Subtheme 1.2 The Osteoarthritic Shoulder

All PILs provided some explanation about shoulder pathology, and this was predominantly related to osteoarthritis (OA). The dominant explanatory model was ‘wear and tear’, or the ‘worn’ or ‘damaged’ shoulder.

Arthritis of the shoulder gradually wears down the cartilage covering the bones over time… This can cause severe pain and stiffness… (PIL 5)

Some PILs suggested that as articular cartilage is worn away, the underlying bones become exposed and rub together:

Following damage to the cartilage the surfaces can become roughened and inflamed leading to pain and stiffness. This can lead to a grating sensation known as crepitus with shoulder movements. (PIL 34)

These descriptions risk undermining the perceived value of any treatment options that do not involve replacing the ‘worn’ components of the shoulder.

It was acknowledged within a small number of PILs that OA and rotator cuff tears are common age‐related changes; however, no PIL explained that these findings are prevalent in the asymptomatic population or that they are not strongly associated with symptoms.

Arthritis of the shoulder most commonly refers to osteoarthritis, or “wear & tear” arthritis. This is often a gradual process in which the normal cartilage covering on the ball (humeral head) and socket (glenoid) of the shoulder joint becomes worn. This can lead to uncovering of the underlying bone and a stiff, painful shoulder joint. (PIL 13)

Sometimes the deep layer of muscles (the ‘Rotator Cuff’) which help control shoulder movements can also be worn or damaged. (PIL 10)

In keeping with the biomedical model, PILs tended to inform the reader that OA is progressive and, therefore, that symptoms will inevitably worsen over time, rather than explaining that the clinical course of OA is variable and not necessarily progressive.

…The more the arthritis advances, the sooner the pain occurs, ultimately even at rest and night. When arthritis becomes advanced, patients mostly suffer with constant severe pain, stiffness and swelling… (PIL 5)

When describing the arthritic shoulder joint, medical jargon was common across the PILs:

The diagnosis usually involves radiographic imaging with an Xray possibly showing osteophytes (bone growths) around the joint. These bone growths occur in response to the damage but rather than a healing effect they often have a derisory effect. Another form of arthritis is known as Rheumatoid arthritis (RA). RA is a systemic inflammatory disorder that affects the capsule around synovial joints. (PIL 34)

### 3.4 Theme 2: Surgery as the Optimum Treatment

Although all PIL provided some explanation of the clinical indications for shoulder replacement surgery, only a proportion discussed alternative treatment options or other factors to consider in the decision‐making process. Surgery was predominantly portrayed as the optimum treatment.

### 3.5 Subtheme: 2.1 Alternatives to Surgery

Some PIL discussed non‐surgical treatments including medication, physiotherapy, and steroid injections, but consistently, these were portrayed as suboptimal treatment options.

Alternative treatments such as pain killers and physiotherapy can help, but these treatments will not stop your condition from worsening. (PIL 7)

Non‐surgical treatments were predominantly discussed within the context that they will have previously failed:

You may have undergone a regime of conservative measures such as painkillers, injections, exercise and physiotherapy to help improve your pain and function. However, if these have failed, shoulder replacement surgery can be recommended. (PIL 5)

### 3.6 Subtheme 2.2 Surgical Decision Making

There was conflicting advice between PIL with regard to surgical decision making. Some PILs advised that shoulder replacement surgery should only be considered when symptoms are significantly affecting quality of life and that the decision to have surgery should be shared between the patient and surgeon:

A shoulder replacement is normally seen as a last resort… (PIL 3)

The decision to proceed with an operation is an individual choice between every patient and their surgeon. You will only be offered an operation if your surgeon believes that this will help improve your symptoms. Very few operations are essential, and all have a degree of risk… (PIL12)

In contrast to the idea that very few surgeries are essential, some PIL adopted a more paternalistic narrative in which it was implied that the decision to undergo surgery is that of the surgeon rather than the patient:

Your orthopaedic surgeon has deemed it necessary for you to have an operation called a Total Shoulder Replacement (TSR) (PIL 21)

Furthermore, one PIL explicitly stated that the patient's symptoms risk worsening without shoulder replacement surgery, which could be misleading:

…If the joint is not replaced or resurfaced your condition can become worse. You may have more pain and less movement. (PIL 7)

## 4 Discussion

This study explored the content and language used within publicly available PILs for people considering shoulder replacement surgery and considered how this might influence decision making. PIL content was variable, predominantly within a biomedical framework, and often not conducive to shared decision‐making.

Many PILs described ‘normal’ shoulder anatomy which may risk inferring that the shoulder must be without defect in order to function without pain. This may have implications for patients in terms of contextualising imaging findings and selecting treatment options, given that non‐surgical treatments cannot change the ‘worn’ joint. Developers of PILs need to consider the potential unintended consequences of describing a ‘normal’ joint without appropriate context, including that a ‘worn’ joint can be asymptomatic (Oo and Linklater 2024).

The osteoarthritic shoulder was described in the majority of PILs using the terms ‘wear and tear’, ‘worn’ or ‘damaged’. Previous research highlights that healthcare professionals often use the term ‘wear and tear’ to describe OA as it is deemed to be less threatening and easily understood by patients; however, patients often interpret this term to mean that the body is breaking down and the condition is progressive (Barker et al. 2014; Darlow et al. 2018). These beliefs risk the development of avoidant behaviours as patients are fearful of causing further joint damage (Darlow et al. 2018; Gunn et al. 2017; Robertson et al. 2017). Patients may therefore be reluctant to undertake exercise for fear that it may exacerbate their condition (Barker et al. 2014; Darlow et al. 2018), despite this being a core treatment for OA (NICE 2022). Patients may also believe that joint replacement will eventually be required (Cronström et al. 2019). Similar findings have been observed in subacromial pain and rotator cuff tear populations, where the belief that surgery is necessary to fix the structural issue has been highlighted (Cuff and Littlewood 2018; Moffatt et al. 2024).

Developers of PILs could address these beliefs by explaining that OA is not always progressive (NICE 2022) and that symptoms are influenced by multiple biopsychosocial factors (Ching et al. 2023; Kouraki et al. 2022; NICE 2022; Overton et al. 2024). While several PILs acknowledged that OA and rotator cuff tears are common in older populations, it may be helpful to also note that structural changes seen on imaging are often present in asymptomatic individuals (Oo and Linklater 2024). Additionally, it may also be useful to highlight that structural abnormalities have only a low to moderate correlation with pain and function across various MSK conditions (El‐Tallawy et al. 2021), including shoulder OA (NICE 2022).

It is recommended that individuals accessing healthcare services be involved in developing health education materials to ensure they meet the needs of the target audience (McDonald et al. 2023). Health information is more effective and easier to understand when written from the patient's perspective, avoiding jargon and unnecessary acronyms (Findeis and Patyk 2020). While our review of the PILs did not formally analyse the average reading age of the text, studies show that health education materials are often written above the average adult reading level (Oliffe et al. 2019; Rooney et al. 2021). In the UK, 7.1 million adults read at or below the level of a typical nine‐year‐old (Powell 2022), underscoring the need to consistently write using plain language and avoid technical jargon. Co‐producing PILs with patient representatives, carers, and clinicians would help ensure these materials are accessible and fit for purpose (Price et al. 2021).

Developers of future PILs should consider including a balanced overview of all available treatment options to support shared decision‐making (NICE 2021). Our findings suggest that many PILs imply that joint replacement surgery is the optimal, and sometimes the only, treatment for people living with shoulder OA, which is not the case (NICE 2022). Additionally, PILs often fail to present the natural progression of the condition as a viable alternative for some individuals. Involving a diverse group of stakeholders, including patient and carer representatives, non‐surgical clinicians, and surgeons, in the development of future PILs could help ensure that these materials provide a more balanced perspective.

## 5 Limitations

This study was conducted using publicly available shoulder replacement PILs published by UK NHS Trusts and involved an extensive online search. Analysis was conducted on 38 PIL; however, it is acknowledged that many of the UK's NHS Trusts do not have published PILs available online ; therefore, our findings may not be representative of these Trusts. Nonetheless, included PILs were produced by NHS trusts across a variety of geographical locations, which may improve the transferability of the results.

It is recognised that PIL are only one of many potential sources of information, and therefore, it is unlikely that patients' decision‐Os making will be based solely on the information provided within them. It has been reported that clinical consultations are the most important source of information for patients (Oedekoven et al. 2019); however, these discussions were not analysed within the current study. Additionally, neither patients nor members of the public were involved in the data analysis process, representing a further limitation.

It is acknowledged that the authors' background, beliefs and experiences will have influenced the analysis of the data within this study (Olmos‐Vega et al. 2022). As a research team comprising clinical and academic physiotherapists with a focus on shoulder conditions and shared decision‐making, our perspectives may have influenced the emphasis placed on certain aspects of the PILs. It is recognised that this is a feature and not a limitation of the qualitative approach taken within this study (Dodgson 2017; Wilson et al. 2022).

## 6 Conclusion

PILs can play an important role in providing patients with health information. However, the language used to describe the clinical indications for surgery predominantly adhered to a biomedical framework and used terms that have been identified in previous research to be interpreted negatively by patients. Surgery was often presented as the optimal treatment, with insufficient attention given to alternative options. Despite recommendations to incorporate the patient perspective into health education materials, this appeared to be absent in the PILs included.

The findings of this study highlight an opportunity to enhance the quality of health education materials for individuals considering shoulder replacement surgery. Improvements should focus on ensuring that the content and language are accessible, culturally sensitive, and capable of facilitating shared decision‐making.

## Author Contributions

All authors (M.M., N.C., C.L.) were involved in the conception of the research question. Google searches were performed by two authors (N.C. and C.L.). All authors (M.M., N.C., C.L.) were involved in data extraction, data analysis, and drafting of the final manuscript. All authors approved the final version of the manuscript before submission.

## Conflicts of Interest

The authors declare no conflicts of interest.

## Supporting information

Supporting Information S1

## Data Availability

This study used data from publicly available patient information leaflets published online by UK NHS Trusts.
